# Improving analysis of the vaginal microbiota of women undergoing assisted reproduction using nanopore sequencing

**DOI:** 10.1007/s10815-022-02628-4

**Published:** 2022-10-12

**Authors:** Theresa Lüth, Simon Graspeuntner, Kay Neumann, Laura Kirchhoff, Antonia Masuch, Susen Schaake, Mariia Lupatsii, Ronnie Tse, Georg Griesinger, Joanne Trinh, Jan Rupp

**Affiliations:** 1 Institute of Neurogenetics, University of Lübeck, Lübeck, Germany; 2 Department of Infectious Diseases and Microbiology, University of Lübeck, Lübeck, Germany; 3 Department of Gynaecological Endocrinology and Reproductive Medicine, University Hospital of Schleswig-Holstein, Campus Lübeck, Lübeck, Germany; 4 German Center for Infection Research, Partner Site Hamburg-Lübeck-Borstel-Riems, Lübeck, Germany

**Keywords:** Assisted reproduction, In vitro fertilization, Microbiome, Third-generation sequencing, Clinical decision-making

## Abstract

**Purpose:**

Subclinical alterations of the vaginal microbiome have been described to be associated with female infertility and may serve as predictors for failure of in vitro fertilization treatment. While large prospective studies to delineate the role of microbial composition are warranted, integrating microbiome information into clinical management depends on economical and practical feasibility, specifically on a short duration from sampling to final results. The currently most used method for microbiota analysis is either metagenomics sequencing or amplicon-based microbiota analysis using second-generation methods such as sequencing-by-synthesis approaches (Illumina), which is both expensive and time-consuming. Thus, additional approaches are warranted to accelerate the usability of the microbiome as a marker in clinical praxis.

**Methods:**

Herein, we used a set of ten selected vaginal swabs from women undergoing assisted reproduction, comparing and performing critical optimization of nanopore-based microbiota analysis with the results from MiSeq-based data as a quality reference.

**Results:**

The analyzed samples carried varying community compositions, as shown by amplicon-based analysis of the V3V4 region of the bacterial *16S rRNA gene* by MiSeq sequencing. Using a stepwise procedure to optimize adaptation, we show that a close approximation of the microbial composition can be achieved within a reduced time frame and at a minimum of costs using nanopore sequencing.

**Conclusions:**

Our work highlights the potential of a nanopore-based methodical setup to support the feasibility of interventional studies and contribute to the development of microbiome-based clinical decision-making in assisted reproduction.

**Supplementary Information:**

The online version contains supplementary material available at 10.1007/s10815-022-02628-4.

## Introduction

The microbiome of the human vagina has deep implications on women’s sexual health by controlling sexually transmitted infections [1, 2] and thereby affecting the female fertility as well [3–5]. Recent advances identified a direct association between the urogenital microbiota and the fertility status of women. Thus, women with endometriosis have been found to have a cervicovaginal microbial composition distinguishable from other women [6], while the vaginal microbiota have been shown to distinguish women with infectious infertility from infertile women with non-infectious causes [3]. It has already been a few years since Moreno et al. suggested that the composition of the microbiota of the urogenital tract of women may have implications on fertilization success and pregnancy [7]. The likelihood of achieving a pregnancy after embryo transfer has been shown to be linked to specific microbiota of the vagina [8], with lactobacilli identified as a promoting factor [9]. However, which specifics are beneficial for treatment success in ART is still under debate [10]. Thus, *Lactobacillus (L.) iners* has been described as predictive of treatment success and suggested as a marker supporting the start of an in vitro fertilization (IVF) trial by Koedooder et al. (2019) in contrast to postponing treatment in case of an unfavorable microbiota composition is present. More recently, *Lactobacillus gasseri* was associated with successful IVF treatment [11]. Congruently, non-*Lactobacillus* species are described as non-favorable for the likelihood of achieving a pregnancy after embryo transfer [5, 7, 9, 11].

As given, it appears evident that infertility, the success of its treatment via ART, and the microbial composition are interconnected. Therefore, new, fast, and reliable methods are required to help clinicians decide whether to initiate treatment in a given subject at a given time point. Various methods are currently available for analyzing the microbiota composition based on amplicons or metagenomics sequencing. While metagenomics sequencing is the more elaborate method to describe the microbial composition in depth [12], amplicons are a good and economical approximation of the microbiota if applied accurately [13, 14]. From the perspective of clinical utility, the most used sequencing technique, MiSeq Illumina sequencing, has the disadvantage of long-running sequencings (several days) and high costs and, therefore, is impracticable for small sample sizes. With increased use, nanopore third-generation sequencing is becoming an alternative or complement to next-generation sequencing and has been successfully applied for sequencing nasal and gut microbiota before [15, 16]. Specifically, nanopore sequencing has been determined advantageous for clinical laboratories due to its low cost, rapid turnover times, and limited need of bioinformatic skills [17, 18].

In this line, studies and attempts to analyze the urogenital microbiota have meanwhile also been using nanopore sequencing approaches (e.g., [19, 20]) and nanopore sequencing was stated to be reliable for vaginal [21] and endometrial [22] microbiota analysis. However, those studies lack coverage of the wide variety of the microbial communities in its entirety previously described to exist in the human female urogenital tract, leaving open questions about the precision of the methodical setups.

## Objective

We here aim to compare and optimize several strategies for analyzing the cervicovaginal microbiota of patients undergoing in vitro fertilization using the MinION Nanopore sequencer. In doing so, we set a specific focus on covering a broad spectrum of varying vaginal microbial communities. In order to validate our results, we compare the performance of our new strategies to the widely used (e.g., [23–26]) and recommended [13, 27] amplicon-based microbiota estimation utilizing the hypervariable V3/V4 regions of the bacterial *16S rRNA gene* using MiSeq sequencing.

## Methods

### Patients and ethics

The vaginal swab samples analyzed herein stem from an ongoing study on microbiome influence on IVF success at the University Hospital of Schleswig–Holstein, Germany. The ten samples analyzed within this manuscript were chosen from the large cohort with a focus on encompassing a wide range of different community state types and bacterial communities’ variances. No other criteria were applied when selecting samples.

### Sampling

Swabs (Copan UTM®; Universal Transport Medium for viruses, chlamydia, mycoplasma, and ureaplasma) were taken from the posterior fornix of the vagina under speculum visualization. The swab was taken not to be contaminated by contact with the vulva, the speculum, or the gloves of the physician. Swabs were frozen and stored at − 80 °C directly after sampling.

### DNA isolation

Swabs were vortexed at highest speed for 1 min. DNA was isolated from 250 µl of the remaining buffer using the Qiagen DNeasy PowerSoil® Kit, following the instructions from the manufacturer’s protocol. Isolated DNA was stored at − 20 °C. We did homogenization after adding the sample and solution C1 using a precellys24 device (Peqlab) at 5000 rpm for 15 s.

### Amplicon generation

We used several strategies for generating amplicons for sequencing on different systems. First, amplicons were generated for Illumina short-read sequencing. Therefore, we amplified the V3/V4 region (V3F primer: 5′-CCTACGGGAGGCAGCAG-3′/V4R primer: 5′-GGACTACHVGGGTWTCTAAT-3′) of the bacterial *16S rRNA gene* sequences from each isolated sample. All primers contained unique identifier sequences (barcodes) to distinguish between the samples following published methods [28, 29] with an optimized primer design [13] (see Supplementary Table 1 and Supplementary Table 3 for complete primer sequences and PCR conditions).

Subsequently, we generated amplicons for nanopore sequencing. Enabling the use of longer reads obtained from nanopore sequencing, we used primer pairs spanning a longer sequence of the *16S rRNA* gene. First, we prepared a nearly full-length *16S rRNA gene* amplicon (27F-YM primer: 5′- AGAGTTTGATYMTGGCTCAG -3′/1492R-Y primer: 5 ′- GGTTACCTTGTTAYGACTT -3′; see Supplementary Table 2 for complete primer sequences) as previously described [30, 31]. Secondly, we prepared shorter amplicons using the above-described V3F forward primer and the same 1492R-Y reverse primer. As a comparison, we processed a subset of *n* = 4 samples with the Nanopore *16S rRNA gene *Barcoding Kit (SQK-RAB204), following the manufacturer’s instructions. The *16S rRNA gene* Barcoding Kit includes the primers 27F-M and 1429R (https://nanoporetech.com/).

### Sequencing

We sequenced the V3/V4 region amplicons on a MiSeq sequencer (Illumina) using the MiSeq Reagent Kit v3 (600 cycles). Longer amplicons of the same samples were sequenced on a MinION (Oxford Nanopore Technologies). The nanopore amplicon sequencing was performed using the Nanopore Rapid PCR Barcoding Kit (SQK-RPB004), following manufacturer’s instructions. The kit provides unique barcodes that allow multiplexing samples and sequencing them in parallel.

### Data processing

For Illumina sequences, the raw data were processed using mothur [32] version 1.44.1, following the mothur SOP available online (https://mothur.org/wiki/miseq_sop/) as we did in our recent studies (e.g., [33, 34]). In brief, we removed all sequences with ambiguous bases, a length greater than the amplified fragment, and a homopolymer length greater than 12. The sequences were aligned with the SILVA reference database [35, 36] and chimeras were removed using the vsearch algorithm [37]. We clustered the processed sequences into operational taxonomic units (OTU) with a global identity threshold of 97% or performed classification-based analysis. The species-level classification was performed using STIRRUPS [38]. To evaluate the nanopore amplicon sequences, we ran the same methodology as mentioned above, though adapting the data processing to the respective length of the amplicons.

### Statistical analysis

The statistical analysis and graphical visualizations were performed in R [39] version 4.0.3. Following a comparison of the read fraction of taxa, Shannon’s and Simpson’s index was calculated with vegan [40] based on OTU to measure alpha diversity and compare different sequencing approaches. The Friedman test was performed to test for significance, and if significance was reached, pairwise comparisons using Wilcoxon signed rank test with Bonferroni correction were performed. Distance analysis and principal coordinates analysis were carried out based on Bray–Curtis distances of the different samples to measure the beta diversity. Utilizing the R package BoutrosLab.plotting.general [41], heatmaps were created. The clustering was performed using the read fraction of the most abundant taxa and the sequencing approach was included as a covariate.

## Results

Here, we aim to test the reliability of Nanopore MinION sequencing as an alternative to the current methods of microbiota sequencing on ten selected vaginal swabs of women undergoing in vitro fertilization. We, therefore, compared the microbial composition as shown by Illumina sequencing using the V3V4 hypervariable regions with three amplicon strategies for nanopore sequencing (Supplementary Fig. 1).

### Nanopore sequencing requires improved primer choice to reflect bacterial community composition

**Fig. 1:**
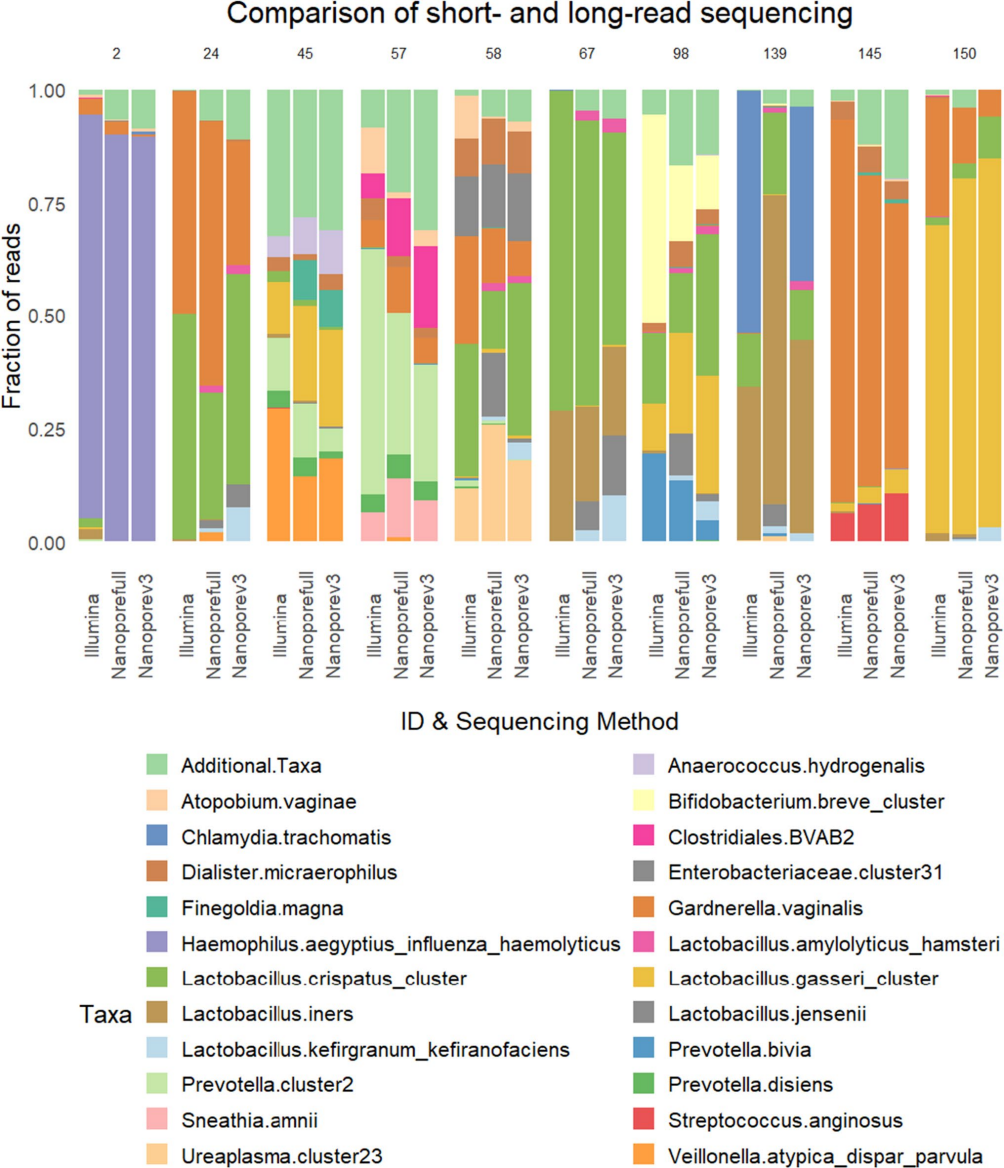
Comparison of classified species using short- or long-read sequencing approaches. The stacked bar plot of the fraction of reads shows the most abundant bacterial taxa classified from the *16S rRNA gene* amplicons. The sample IDs are indicated on the top and the sequencing approaches are on the bottom *x*-axis. Illumina: Illumina short-read sequencing (V3F/V4R), Nanoporefull: nanopore long-read sequencing (27F-YM/1492R-Y), Nanoporev3: nanopore long-read sequencing (V3F/1492R-Y)

As a first approximation to assess the comparability of nanopore and Illumina sequencing results, we choose samples representing a wide range of different community state types [42] and bacterial communities. This includes samples dominated by different *Lactobacillus* species and samples dominated by species known to influence vaginal health (e.g., *Bifidobacterium breve*, *Chlamydia trachomatis*, *Gardnerella vaginalis*, *Sneathia amnii*) (Fig. 1). After preliminary evaluating a subset of samples, the *16S rRNA gene* Barcoding Kit does not recapitulate Illumina sequencing results by using the universal primer pair 27f/1429R (Supplementary Fig. 1). Hence, we switched to a custom-made degenerated 27f-YM forward and 1429R-Y reverse primer for amplicon generation. Based on this, we compared relative fractions of taxonomic reads between different sequencing strategies. By this approach, we show that all but one major taxon from Illumina sequencing are recapitulated by nanopore sequencing. The exception was *C. trachomatis*, displaying approximately 50% of reads assigned to sample 139 in Illumina sequencing, which was missing when the degenerated 27f-YM primer was used in nanopore sequencing (Nanoporefull). In contrast, nanopore sequencing using the V3F primer (as used for Illumina sequencing, Nanoporev3) captures a comparable fraction of *C. trachomatis* reads as Illumina sequencing (Fig. 1, sample number 139).

### Dissimilarity-based comparison of the sequencing approaches

**Fig. 2:**
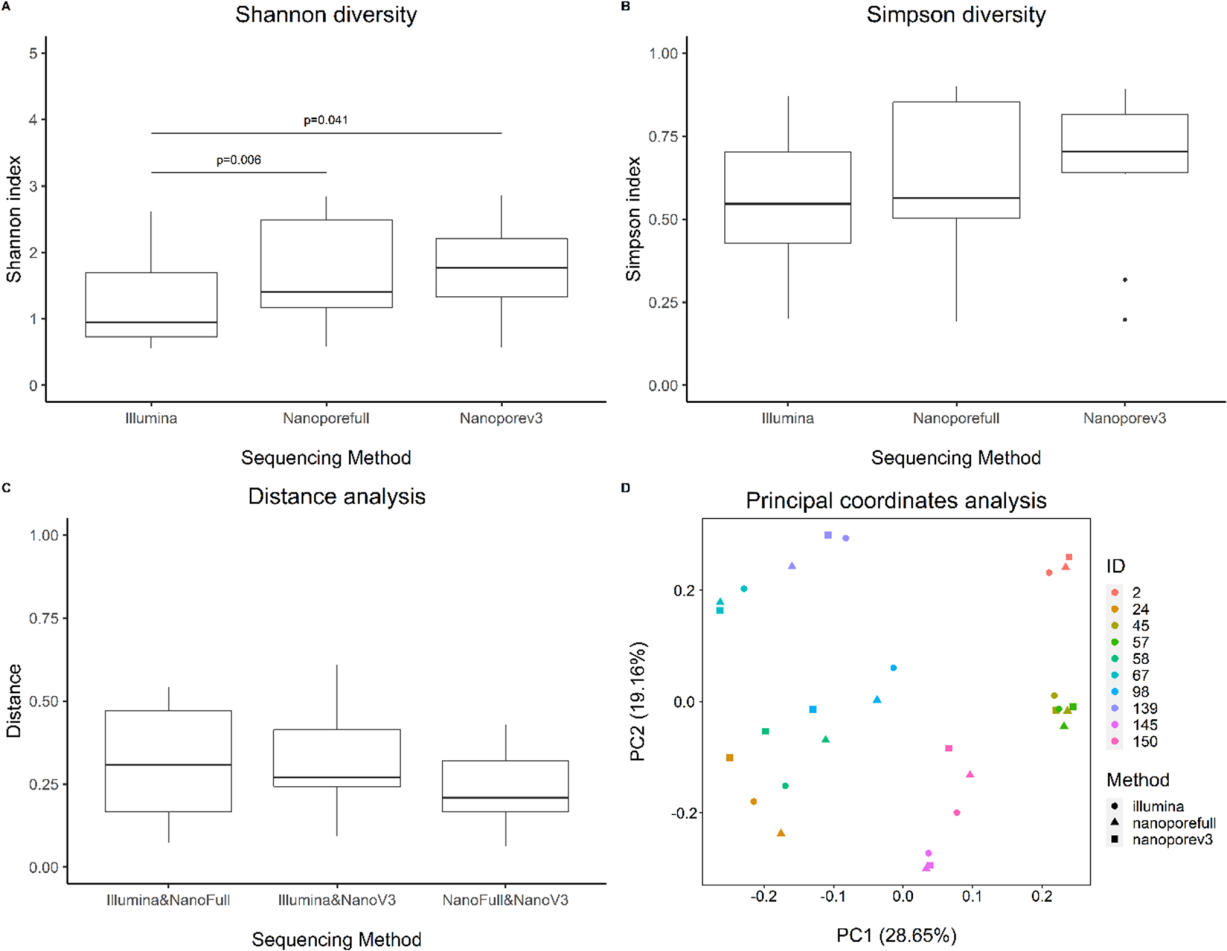
Diversity and similarity of bacterial taxa resulting from short- or long-read sequencing approaches. Boxplots showing the Shannon (**A**) and Simpson diversity index (**B**). **C** Boxplots showing the Bray–Curtis distances between the samples analyzed with short- or long-read sequencing approaches. **D** Principal coordinate analysis based on Bray–Curtis distances of the different samples, as indicated by color. The sequencing method is indicated by shape. Illumina: Illumina short-read sequencing (V3F/V4R), Nanoporefull: nanopore long-read sequencing (27F-YM/1492R-Y), Nanoporev3: nanopore long-read sequencing (V3F/1492R-Y). Statistics for panels **A**–**C** were carried out using Friedman test, followed by pairwise Wilcoxon signed rank test if Friedman was displaying *p* < 0.05

We compared *alpha* and *beta* diversity measures in order to assess how similar the results of different sequencing strategies are. While Shannon’s diversity index was indicated to differ between sequencing approaches (Fig. 2A, Friedmann test; *p* = 0.002, post hoc test results given in the figure), we could not identify significant differences between sequencing strategies when using Simpson’s diversity indices (Fig. 2B). We further compared distances between samples based on Bray–Curtis dissimilarity index. Stratifying for the distance between samples from different sequencing strategies, no significant differences are apparent (Fig. 2C). Last, we ran principle coordinates analysis for the entire data set and observed that strategies cluster together for each sample of this study (Fig. 2D).

### Microbiome samples from the same individual cluster together independent of the sequencing approach

**Fig. 3:**
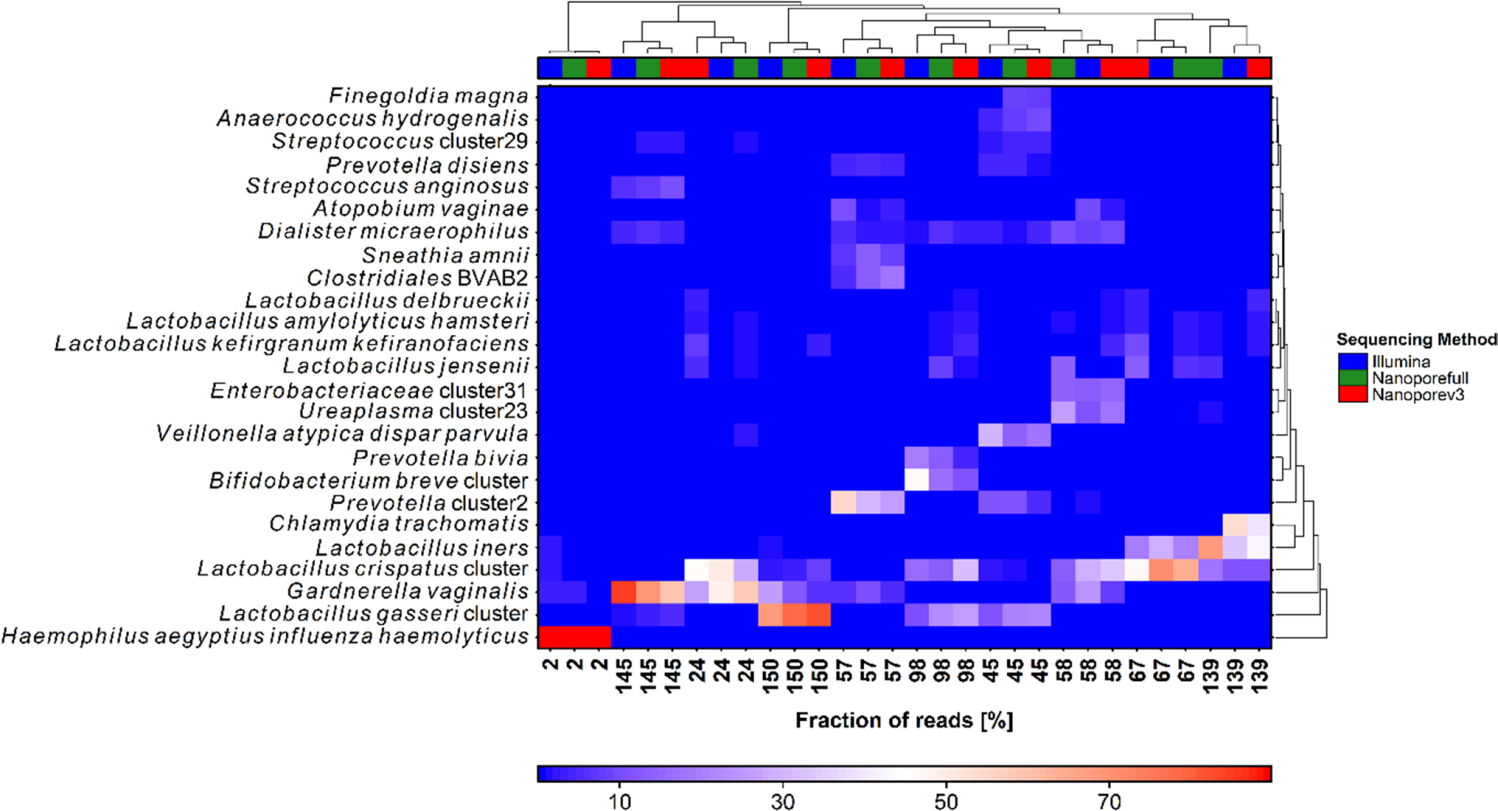
A heatmap based on the fraction of reads assigned the most abundant bacterial taxa. Clustering of the taxa into the different sequencing methods was included, i.e., Illumina: Illumina short-read sequencing (V3F/V4R), Nanoporefull: nanopore long-read sequencing (27F-YM/1492R-Y), Nanoporev3: nanopore long-read sequencing (V3F/1492R-Y)

To clearly visualize the similarity of the different sequencing strategies, we utilized a heatmap plot with hierarchical clustering of community structure based on the relative fraction of reads (Fig. 3). We also show in this case that for all samples, the different sequencing strategies clustered together. However, the length of the dendrogram arms indicates that for sample 139, the distance to the other sequencing strategies of the full-length nanopore sequencing (Nanoporefull), lacking *C. trachomatis* sequences, is large.

## Discussion

It has been suggested that the focus of research on the human microbiome will shift from studies describing the function of the microbiome toward studies employing the microbiota for clinical applications, such as using it as a diagnostic marker [43]. Though the awareness of the importance of the microbiome as an indicator of human health has increased drastically over the last decade, the lack and need for clinical test strategies are still evident [44]. The widely used standard in microbiota analysis from a position of throughput and costs is amplicon-based microbiota analysis [45], primarily using Illumina MiSeq sequencing. This method has great value, especially through the precise and parallel sequencing of a large number of samples. However, Illumina MiSeq sequencing lacks scalability and involves time-consuming procedures, complicating rapid data production. In contrast, due to its scalability, the MinION sequencer runs smaller sample numbers but can produce microbial community profiles within 24 h for individual samples [46].

In reproductive health, the role of the vaginal microbiome for fertility has been assessed [3, 7, 9, 47] and it appears that the microbial composition can add valuable information to the prediction of ART treatment success versus failure [5, 11, 48, 49]. Some previous studies have suggested nanopore sequencing as a reliable methodical solution to determine vaginal and urogenital microbiota in a clinical setting [19–22]. This study focuses on optimizing strategies to provide a fast and reliable method for describing the broad variety of vaginal microbiota of individual samples in women undergoing in vitro fertilization. As tested first, the *16S rRNA gene *Barcoding Kit provided by the company does not provide a sufficient resolution as it fails to identify central bacterial taxa such as *Gardnerella vaginalis* and *Bifidobacterium* (Supplementary Fig. 1). This problem is known to be addressed to the classical 27F primer used by a significant fraction of microbiota studies. It has been shown to fail to identify *Chlamydia trachomatis* as well [13, 31]. Therefore, as suggested before, we have subsequently used a degenerated version of this forward primer (27F-YM) to circumvent this issue [31]. We managed to recapitulate the data produced by the MiSeq sequencing more closely with this approach. However, *C. trachomatis* still was not detected even with the degenerated 27F-YM primer within the nanopore sequencing data. Particularly, in the given case, it overestimates *L. iners* when using the Nanoporefull version. This bias is important to consider, as Koedooder et al. suggested a high fraction of *L. iners* to increase the success chance of embryo implantation [5]. Noteworthy, the respective patient faced a negative outcome from in vitro fertilization (Supplementary Table 4). To achieve higher precision with nanopore sequencing, we used the V3F primer [28, 29] as we did with short-read sequencing [3, 13] instead of the 27F-YM forward primer. Using this setup, we optimized the recapitulation of the microbial community for all samples tested, showing that the use of the MinION third-generation sequencer describes the microbial pattern of vaginal bacterial communities with high accuracy. We want to point out that further improvements to the precision of the herein described methodology may be possible by incorporating the very recently published Emu-algorithm [50]. This approach has been developed for species level classification from Nanopore data and been described to reduce false positive classifications in long-read data [50].

In conclusion from our study, adapting amplicon-based microbiota sequencing of the human vagina on a MinION sequencer provides precise community analysis and a time and cost benefit, which may be relevant for clinical decision making, as it has been pointed out before [17, 18]. Of note, the samples from women with a positive pregnancy test following an embryo transfer (samples 45, 67, and 98; Supplementary Table 4) were colonized with either *L. gasseri* or *L. iners* (Fig. 1). Both species have been acknowledged to be positively correlated with treatment success [5, 11]. While we still assume a gap of knowledge in how *lactobacilli* contribute to vaginal health [51], replenishing *Lactobacillus* species—as suggested recently [11]—may become a solution to enhance the success of in vitro fertilization. Opening ideas about the diagnostic basis for such planning, we hope to provide a critical methodical step toward using microbiota as a parameter in clinical decision-making.

## Supplementary Information

Below is the link to the electronic supplementary material.

Supplementary file1 (DOCX 201 KB)

## Data Availability

The raw sequencing data can be found at the European Nucleotide Archive under accession number PRJEB53337.
