# Supplementary material for: Improving analysis of the vaginal microbiota of women undergoing assisted reproduction using nanopore sequencing
Source: J Assist Reprod Genet. 2022 Oct 12;39(11):2659–67. doi: 10.1007/s10815-022-02628-4 (PMC9722992; doi:10.1007/s10815-022-02628-4)
Supplement: Supplementary file 1 — Supplementary file1 (DOCX 201 KB) [file 10815_2022_2628_MOESM1_ESM.docx]

**Supplementary Table 1: Overview of PCR primer sequences Illumina sequencing**

| **Sequencing approach** | **Forward/Reverse primer name** | **Primer sequence^a^** |
| --- | --- | --- |
| Illumina sequencing | Forward: V3F | 5′-AATGATACGGCGACCACCGAGATCTACAC **ATCGTACG *ACACTCTTTCCCTACACGACGCTCTTCCGATCT***  CCTACGGGAGGCAGCAG-3′ |
|  | Reverse: V4R-A | 5′-CAAGCAGAAGACGGCATACGAGAT **AACTCTCG *GTGACTGGAGTTCAGACGTGTGCTCTTCCGATCT*** GGACTACHVGGGTWTCTAAT-3′ |
|  | Reverse: V4R-B | 5′-CAAGCAGAAGACGGCATACGAGAT **ACTATGTC *GTGACTGGAGTTCAGACGTGTGCTCTTCCGATCT A*** GGACTACHVGGGTWTCTAAT-3′ |
|  | Reverse: V4R-C | 5′-CAAGCAGAAGACGGCATACGAGAT **AGTAGCGT *GTGACTGGAGTTCAGACGTGTGCTCTTCCGATCT TC*** GGACTACHVGGGTWTCTAAT-3′ |
|  | Reverse: V4R-D | 5′-CAAGCAGAAGACGGCATACGAGAT **CAGTGAGT *GTGACTGGAGTTCAGACGTGTGCTCTTCCGATCT CTA*** GGACTACHVGGGTWTCTAAT-3′ |
|  | Reverse: V4R-E | 5′-CAAGCAGAAGACGGCATACGAGAT **CGTACTCA *GTGACTGGAGTTCAGACGTGTGCTCTTCCGATCT GATA*** GGACTACHVGGGTWTCTAAT-3′ |
|  | Reverse: V4R-F | 5′-CAAGCAGAAGACGGCATACGAGAT **CTACGCAG *GTGACTGGAGTTCAGACGTGTGCTCTTCCGATCT ACTCA*** GGACTACHVGGGTWTCTAAT-3′ |
|  | Reverse: V4R-G | 5′-CAAGCAGAAGACGGCATACGAGAT **GGAGACTA *GTGACTGGAGTTCAGACGTGTGCTCTTCCGATCT TTCTCT*** GGACTACHVGGGTWTCTAAT-3′ |
|  | Reverse: V4R-H | 5′-CAAGCAGAAGACGGCATACGAGAT **GTCGCTCG *GTGACTGGAGTTCAGACGTGTGCTCTTCCGATCT*** GGACTACHVGGGTWTCTAAT-3′ |
|  | Reverse: V4R-I | 5′-CAAGCAGAAGACGGCATACGAGAT **GTCGTAGT *GTGACTGGAGTTCAGACGTGTGCTCTTCCGATCT A*** GGACTACHVGGGTWTCTAAT-3′ |
|  | Reverse: V4R-J | 5′-CAAGCAGAAGACGGCATACGAGAT **TAGCAGAC *GTGACTGGAGTTCAGACGTGTGCTCTTCCGATCT TC*** GGACTACHVGGGTWTCTAAT-3′ |

^a^primer structure: 5′-adaptor to flowcell **unique_identifyer_sequence *sequencing_primer_binding_site*** ***heterogeneity_spacer*** target_binding_site-3′

**Supplementary Table 2: Overview of PCR primer sequences Nanopore sequencing**

| **Sequencing approach** | **Forward/Reverse primer name** | **Primer sequence** |
| --- | --- | --- |
| Nanopore sequencing (full-length 16S rRNA gene PCR) | Forward: 27F-YM | 5’- AGAGTTTGATYMTGGCTCAG -3’ |
|  | Reverse: 1429R-Y | 5‘- GGTTACCTTGTTAYGACTT -3’ |
| Nanopore sequencing (partial 16S rRNA gene PCR) | Forward: V3F | 5′-CCTACGGGAGGCAGCAG-3′ |
|  | Reverse: 1429R-Y | 5‘- GGTTACCTTGTTAYGACTT -3’ |
| Nanopore sequencing (16S Barcoding Kit) | Forward: 27F | 5‘- AGAGTTTGATCMTGGCTCAG -3’ |
|  | Reverse: 1429R | 5‘- CGGTTACCTTGTTACGACTT -3’ |

**Supplementary Table 3: Overview of PCR conditions for the different sequencing approaches**

| **Illumina sequencing (V3F/V4R)** | | |  | **Nanopore sequencing (27F-YM/1492R-Y)** | | |  | **Nanopore sequencing (V3F/1492R-Y)** | | |
| --- | --- | --- | --- | --- | --- | --- | --- | --- | --- | --- |
| 98°C | 00:30 |  |  | 98°C | 50:00 |  |  | 98°C | 50:00 |  |
| 98°C | 00:09 |  |  | 98°C | 00:09 |  |  | 98°C | 00:09 |  |
| 48°C | 01:00 | 30 cycles |  | 52°C | 00:45 | 30 cycles |  | 52°C | 00:30 | 30 cycles |
| 72°C | 01:30 |  |  | 65°C | 01:00 |  |  | 65°C | 01:00 |  |
| 72°C | 10:00 |  |  | 65°C | 05:00 |  |  | 65°C | 05:00 |  |
| 4°C | ∞ |  |  | 4°C | ∞ |  |  | 4°C | ∞ |  |

**Supplementary Table 4.** Outcomes of ART**.**

| **Sample ID** | **Fertility success** |
| --- | --- |
| 2 | negative |
| 24 | negative |
| 45 | positive |
| 57 | negative |
| 58 | negative |
| 67 | positive |
| 98 | positive |
| 139 | negative |
| 145 | negative |
| 150 | negative |

ART= assisted reproduction technology


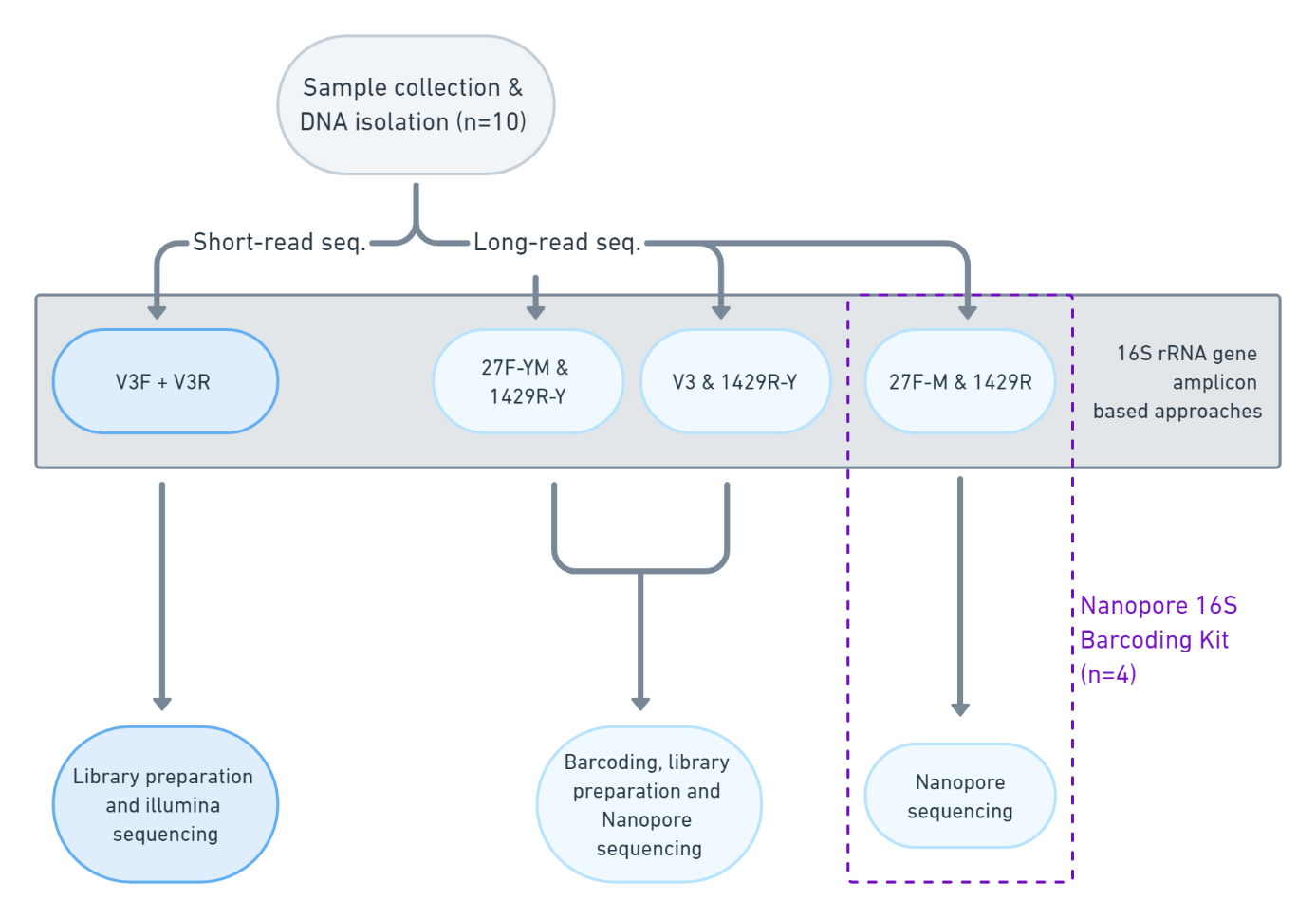


**Supplementary Figure 1. Overview of workflow.** For this study n=10 vaginal microbiome samples were included. For the classification of the bacterial taxa, the 16S rRNA gene was partially or nearly in full-length amplified. The therefore used primer pairs are shown in the grey box. On the one hand, all samples were processed with an established pipeline for Illumina short-read sequencing. On the other hand, all samples were processed with a custom pipeline for Nanopore sequencing, testing two different sets of primers (V3F + 1429R-Y, 27F-YM + 1429R-Y). A subset of n=4 samples was processed with the Nanopore 16S Barcoding Kit as well.

**
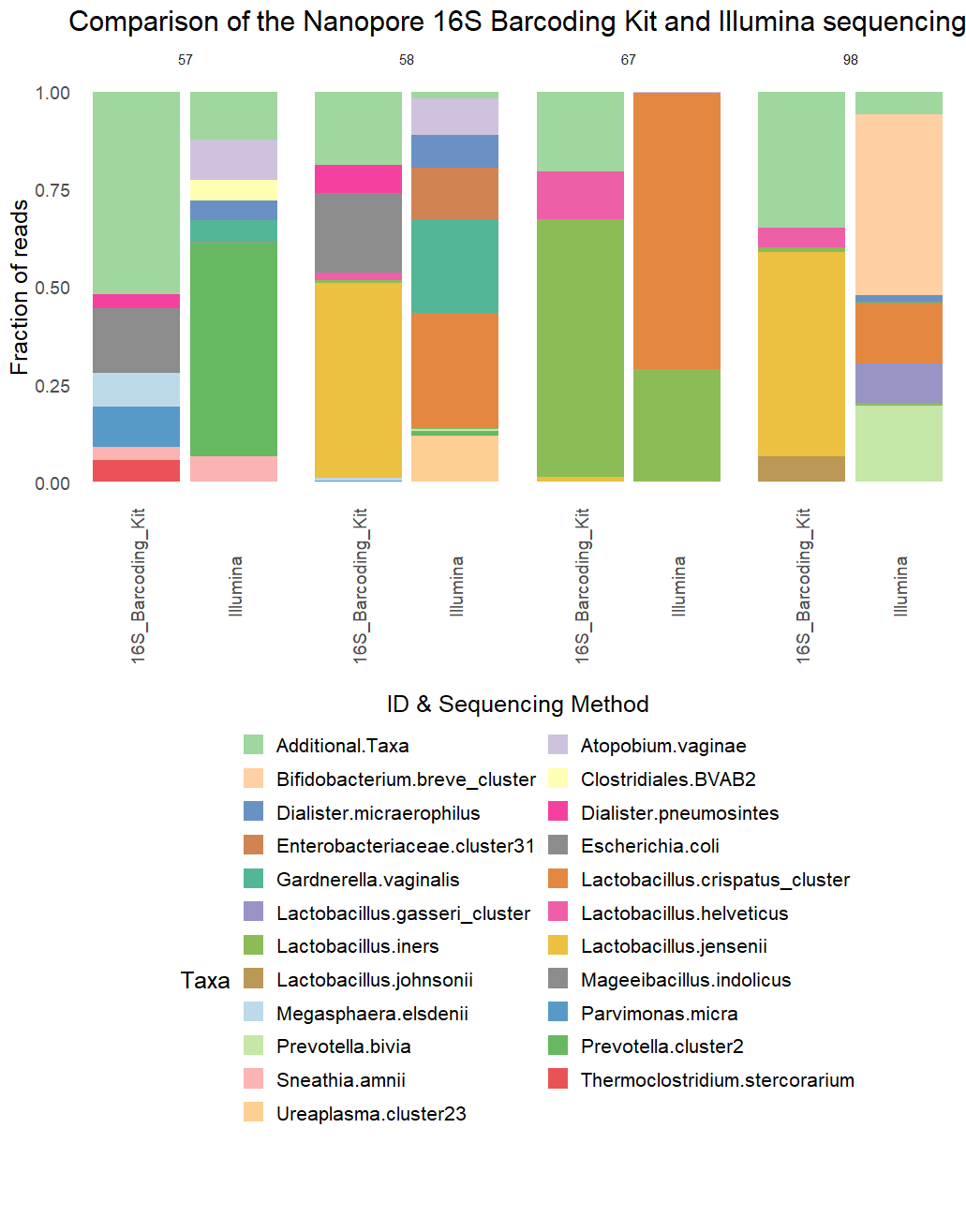
**

**Supplementary Figure 2. Comparison of classified spices using short- or long-read sequencing approaches.** The stacked bar plot of the fraction of reads shows the most abundant bacterial taxa classified from the 16S amplicons. The sample IDs are indicated on the top and the sequencing approaches are on the bottom x-axis.

Illumina = Illumina short-read sequencing (V3F/V4R), 16S_Barcoding_kit = Nanopore long-read sequencing (27F/1492R) included in SQK-RAB204 (<https://store.nanoporetech.com/us/16s-barcoding-kit.html>).
